# O9-3 Reducing workplace sedentary behaviour: An exploration of the facilitators and barriers to professional men reducing prolonged sedentary behaviour

**DOI:** 10.1093/eurpub/ckac094.067

**Published:** 2022-08-29

**Authors:** Gail Nicolson

**Affiliations:** Public Health and Primary Care, Trinity College Dublin, Dublin, Ireland

**Keywords:** Workplace, sedentary behaviour, socioecological model, males, barriers, facilitators

## Abstract

**Introduction:**

There is a growing interest in workplace interventions to reduce sedentary behaviour. Professional males are most at risk of prolonged sedentary behaviour, are considered difficult to reach in terms of recruitment to studies, and may require targeted health promotion interventions. Research on the views of senior managers and executives as well as employees in a professional setting in relatively underdeveloped. Studies have shown that a ?one size fits all' approach is not suitable in behaviour change interventions. The aim of this study was to qualitatively explore professional male workers' facilitators and barriers to reducing workplace sedentary behaviour and their views on suggested intervention strategies to ensure relevance and practicality of the intervention in each specific context.

**Methods:**

Three semi-structured focus groups and one interview were conducted with a purposive sample of 25 professional men (15 employees and 10 senior managers). The men were recruited using convenience sampling from two professional worksites in Dublin, Ireland. Transcripts were analysed using thematic analysis.

**Results:**

The men identified a range of intrapersonal, interpersonal and organisational barriers and facilitators to reducing workplace sedentary behaviour. Perceived facilitators included individual motivation, the formation of new habits likely through small changes, management buy-in and support and organisational culture. Perceived barriers included the primacy of work, job requirements of being desk-bound, and management expectations. Views of suggested strategies for a novel multicomponent intervention to target the determinants of sedentary behaviour as informed by the socio-ecological model were positive overall.

**Conclusions:**

The needs, preferences and opinions of professional men to participate in a workplace intervention to reduce sedentary behaviour should be taken into consideration prior to implementation in a workplace setting. This is important to ensure relevance and the practicality of intervention strategies in the particular settings.

